# Architecture-Specific Performance of Silica and MWCNT Modified Hybrid Epoxy Laminates Under Mechanical Loading and Fracture

**DOI:** 10.3390/polym18202463

**Published:** 2026-10-09

**Authors:** Muhammad Tariq Bashir, Khawaja Atif Naseem, Atif Khan, Muhammad Rehan Sadiq, Md. Munir Hayet Khan, Muhammad Ali Sikandar

**Affiliations:** 1 Department of Civil Engineering, CECOS University of IT & Emerging Sciences, Peshawar 25000, Pakistan; 2 Faculty of Engineering and Quantity Surveying (FEQS), INTI International University, Persiaran Perdana BBN, Nilai 71800, Malaysia; munir.hayetkhan@newinti.edu.my; 3 Traffic Safety Engineer, Arkansas Department of Transportation, 10324 I-30, Little Rock, AR 72209, USA; 4 Department of Civil and Environmental Engineering, University of Louisiana at Lafayette, Lafayette, LA 70503, USA; 5 STV Inc., 5750 Genesis Court, Suite 200, Frisco, TX 75034, USA

**Keywords:** hybrid epoxy laminates, silica nanoparticles, multi-walled carbon nanotubes, architecture-specific response, fracture morphology, punch shear, uncertainty, sustainable buildings, exploratory machine learning

## Abstract

Hybrid fiber-reinforced epoxy laminates integrate macroscale fiber architecture with nanoscale matrix modification. The effects of nanofillers cannot be isolated from laminate architecture when both variables are altered simultaneously. This study independently evaluated silica-modified Kevlar/basalt/alkali-resistant glass (K/B/ARG), silica-modified Kevlar/carbon/alkali-resistant glass (K/C/ARG), and multi-walled carbon nanotube (MWCNT)-modified Kevlar/glass/carbon (K/G/C) laminates. Each laminate family incorporated 0, 1.5, 3.0, 4.5, or 6.0 wt.% nanofiller. Mechanical performance was assessed using tensile, flexural, interlaminar-shear, quasi-static puncture, punch-shear, impact, and energy-absorption tests. Scanning electron microscopy (SEM) was employed to examine fracture morphology, and selected K/C/ARG laminates underwent thermogravimetric analysis (TGA/DTG). For each architecture, 4.5 wt.% nanofiller yielded the highest mean values for most measured responses. At this concentration, K/B/ARG achieved 167.93 ± 5.71 MPa tensile strength, 112.09 ± 3.70 MPa puncture strength, and 1598.1 ± 73.5 J/m Izod impact strength. K/C/ARG achieved 171.95 ± 5.50 MPa tensile strength, 7710 ± 285.3 N puncture load, and 1813.6 ± 79.8 J/m Izod impact strength. K/G/C achieved 215.58 ± 7.76 MPa tensile strength, 28.49 ± 1.20 MPa interlaminar-shear strength, 110.80 ± 5.54 MPa punch-shear strength, and 15.45 ± 0.77 J net energy absorption. Several matrix-sensitive responses declined at 6.0 wt.%, while K/G/C flexural strength remained nearly constant. SEM revealed greater matrix retention and more uniform damage distribution at intermediate nanofiller loading but clustered weak regions at 6.0 wt.%. Single-run TGA indicated a higher principal DTG temperature and greater residue for the 4.5 wt.% K/C/ARG formulation; however, replication is necessary. The convolutional neural network (CNN) analysis was constrained by the limited number of formulation levels, target leakage, and the absence of independent validation. Consequently, 4.5 wt.% is identified as the best-performing tested concentration within each architecture, rather than a universal optimum. Additional durability, fire, and structural-scale testing is required before these laminates can be considered for building design, resilient infrastructure, or sustainable construction.

## 1. Introduction

Fiber-reinforced polymer (FRP) laminates are increasingly utilized in aerospace, transportation, marine, protective, and civil engineering applications [1,2]. These materials offer advantages such as low mass, high specific strength, corrosion resistance, dimensional stability, and ease of repair. Despite these benefits, the brittle behavior of epoxy matrices and limited out-of-plane resistance can lead to matrix cracking, fiber–matrix debonding, and delamination under bending, impact, or localized loading. As a result, laminate performance must be addressed as a multiscale problem. The fiber architecture determines the primary load paths, whereas the matrix and interphase control crack initiation, interlaminar stress transfer, and progressive damage [1,3,4,5,6].

Hybridization involves combining fibers with complementary properties rather than relying on a single type of reinforcement [7,8]. Carbon fibers impart high stiffness and axial load capacity, while Kevlar fibers enhance toughness and energy dissipation. Glass and alkali-resistant glass fibers provide cost-effective stiffness and durability, and basalt fibers offer thermal and environmental resistance. The relative contribution of each property depends strongly on fiber position and orientation within the laminate. Outer and inner plies are subjected to different tensile, compressive, shear, and contact stresses. Therefore, the stacking sequence significantly influences peak load, flexural behavior, impact damage, and delamination [8,9,10,11].

Nanomodification serves as a complement to fiber hybridization by reinforcing resin-rich and interfacial regions. Well-dispersed silica nanoparticles can enhance local matrix stiffness, facilitate stress transfer, deflect cracks, and increase fracture energy. However, excessive silica content raises resin viscosity, which may result in incomplete fiber wetting, particle aggregation, and voids. Consequently, many studies report a non-monotonic response to silica addition. Optimization of silica content must be considered alongside laminate architecture and processing conditions [5,11,12,13,14].

Multi-walled carbon nanotubes (MWCNTs) introduce a distinct nanoscale reinforcement mechanism. Their high aspect ratio enables crack bridging, nanotube debonding, frictional pull-out, matrix constraint, and the development of load-transfer networks. These mechanisms are especially important for interlaminar and localized shear resistance. However, van der Waals forces and nanotube entanglement can lead to agglomerate formation at elevated concentrations. These agglomerates impede resin flow and serve as local stress concentrators. As a result, both experimental and modeling studies identify dispersion quality and processing route as critical factors [15,16,17,18,19].

These observations support a hierarchical framework for interpreting hybrid nanocomposites. Macroscale fibers define the primary stress pathways, while nanoscale modifiers affect matrix cracking, interfacial separation, delamination, and energy dissipation following damage initiation. Mechanical performance cannot be reliably predicted based solely on nominal constituent content. Factors such as fiber orientation, stacking sequence, stiffness mismatch, resin-rich interlayers, and nanofiller distribution can alter the dominant failure mode. Therefore, tensile, flexural, interlaminar, puncture, impact, thermal, and fractographic responses should be evaluated collectively [19,20].

Machine learning offers a means to characterize nonlinear relationships among composition, processing, structure, and properties. However, composite datasets frequently comprise limited independent batches, correlated predictors, and incomplete processing records. A high coefficient of determination derived from a small set of formulations may indicate interpolation within a narrow design space rather than generalizable predictive capability [21,22,23]. Robust evaluation requires leakage-free validation, well-defined data folds, independent batches, uncertainty quantification, physically meaningful input variables, and transparent performance metrics [24,25,26].

This study examines three families of epoxy-based hybrid laminates containing 0, 1.5, 3.0, 4.5, and 6.0 wt.% nanofiller. The laminate systems include silica-modified Kevlar/basalt/AR-glass (K/B/ARG), silica-modified Kevlar/carbon/AR-glass (K/C/ARG), and MWCNT-modified Kevlar/glass/carbon (K/G/C). Their mechanical and morphological responses are evaluated through tensile, flexural, interlaminar shear, quasi-static puncture, punch-shear, impact, energy-absorption, thermal, and scanning electron microscopy (SEM) analyses. This comprehensive assessment investigates the balance between enhanced interfacial reinforcement and defects induced by agglomeration.

The central research question is as follows: How does nanofiller content ranging from 0 to 6.0 wt.% influence the mechanical response and damage morphology of each fixed laminate architecture? It is hypothesized that intermediate nanofiller content will enhance matrix-sensitive properties relative to both unmodified and highest-loading formulations. However, the extent and underlying mechanisms of improvement are expected to be specific to each laminate architecture. This study does not aim to provide a direct causal comparison between silica and MWCNTs, as the nanofiller type, fiber system, stacking sequence, and certain testing procedures differ among the laminate families. Consequently, the convolutional neural network analysis is presented as a secondary descriptive approach limited to the K/G/C–MWCNT dataset.

## 2. Materials and Methods

### 2.1. Materials, Laminate Design, and Fabrication

ARALDITE^®^ LY 556 epoxy resin and ARADUR^®^ HY 951 hardener were combined at a resin-to-hardener mass ratio of 10:1. This material pairing and curing protocol were selected according to the manufacturer’s technical guide and a comparable epoxy-laminate study [27,28]. The reinforcements consisted of woven Kevlar/para-aramid, carbon, E-glass, basalt, and alkali-resistant glass fabrics.

Silica (SiO_2_) nanoparticles were incorporated in Systems I and II, with a nominal diameter of approximately 20 nm and a minimum purity of 99.9% (Anhui Fitech Materials Co., Ltd., Hefei, China; https://www.fitechem.com/category/nano-materials (accessed on 29 September 2026). MWCNTs were utilized in System III, featuring an outer diameter of 10–20 nm, a length of 10–30 μm, and a purity exceeding 95% (Jiangsu XFNANO Materials Tech Co., Ltd., Nanjing, China; https://chinanano.com/collections/n-doped-mwcnts-1 (accessed on 29 September 2026). Nanofiller contents of 0, 1.5, 3.0, 4.5, and 6.0 wt.% were prepared, each calculated relative to the mass of LY 556 resin prior to hardener addition.

The laminate notation designates K for Kevlar, B for basalt, ARG for alkali-resistant glass, C for carbon, and G for E-glass. System I comprised 10 plies arranged as [0°K]_2_/[45°B]_2_/[0°ARG]_2_/[45°B]_2_/[0°K]_2_. System II also contained 10 plies, arranged as [0°K]_2_/[45°ARG]_2_/[0 °C]_2_/[45°ARG]_2_/[0°K]_2_ (Table 1). System III consisted of five plies arranged as [45°K/45 °C/0°G/45 °C/45°K]. The specified angles indicate the fabric orientation relative to the longitudinal axis of the laminate. All laminates exhibited a nominal cured thickness of 4.0 mm. The calculated average cured thickness per ply was 0.40 mm for Systems I and II, and 0.80 mm for System III, determined by dividing the total laminate thickness by the number of plies. These values do not correspond to the measured dry-fabric thickness. A target fiber volume fraction of 45 vol.% was adopted, consistent with comparable hand-lay-up and compression-molding studies of hybrid epoxy laminates [28,29]. The final fiber volume fraction was not experimentally verified by burn-off, digestion, or microscopic image analysis.

For each nano-filled batch, the required mass of filler was incrementally added to the LY 556 resin. The mixture was stirred with a JJ-1 overhead stirrer at 500 rpm for 10 min, followed by probe sonication for 20 min at a nominal frequency of 20 kHz and a rated power of 300 W. A pulsed cycle of 5 s on and 5 s off was employed. The vessel was immersed in an ice-water bath to maintain the resin temperature below 30 °C. The mixing and sonication parameters were selected based on established procedures for particulate-modified epoxy systems [28,30].

Following sonication, HY 951 hardener was incorporated. The mixture was stirred at 300 rpm for 5 min and vacuum-degassed for 10 min. The modified resin was then immediately utilized for fabric impregnation. Pulsed sonication and external cooling ensured the resin temperature remained below 30 °C. Hand lay-up was conducted on a cleaned steel mold coated with a release agent. Each ply was positioned in the specified sequence and impregnated using a brush and roller, with rolling continued until no visible trapped-air pockets remained. The laminate stack was placed between steel platen plates fitted with 4.0 mm spacers and consolidated at 0.50 MPa for 24 h at 25 ± 2 °C. These consolidation parameters are consistent with reported hand-lay-up and compression-molding procedures for epoxy laminates [29,31].

After consolidation, each laminate was demolded and post-cured at 60 °C for 4 h. This post-curing schedule was selected in accordance with the manufacturer’s guidance and published procedures for comparable epoxy laminates [27,28]. The laminates were cooled to room temperature prior to specimen machining.

### 2.2. Specimen Preparation and Mechanical Testing

Thermogravimetric analysis (TGA) and derivative thermogravimetric (DTG, manufactured by Shimadzu Corporation, Shanghai, China) analyses were conducted on K/C/ARG laminates containing 0, 4.5, and 6.0 wt.% silica. A Henven thermogravimetric analyzer (Beijing Henven Experimental Equipment, Beijing, China) was employed. Each specimen, weighing 5.0 ± 0.5 mg, was placed in an open alumina crucible. The heating protocol ranged from 25 to 700 °C at a rate of 10 °C min^−1^ under a continuous nitrogen flow of 50 mL min^−1^. Specimens were prepared as small pieces cut across the full laminate thickness so that each specimen spanned all plies and represented the overall fiber-to-resin ratio. Before each run, the furnace was purged with nitrogen at the measurement flow rate for at least 10 min, and the initial specimen mass was recorded after thermal equilibration at 25 °C. Temperature and mass calibrations were verified with certified Curie-point and calcium oxalate reference standards, and an empty-crucible blank run was subtracted as a baseline correction. The protocol follows ASTM E1131 and ISO 11358-1/-2 practice for the thermogravimetry of plastics. Table 2 summarizes mechanical tests and specimen dimensions used in the experimental program.

A single TGA run was performed for each composition (n = 1). The measured mass was normalized to the initial specimen mass and reported as the remaining mass percentage. The DTG curve was determined as the first derivative of normalized mass with respect to temperature. Degradation-peak temperatures and final residues were obtained directly from the TGA and DTG curves. Due to the single run per composition, standard deviations, confidence intervals, and statistical comparisons were not calculated. Consequently, the thermal results were used exclusively for within-study comparison of the three silica contents. In addition to the degradation-peak temperatures and final residues, the temperature at 5% mass loss (T5%), the tangent-onset decomposition temperature (Td, onset), the temperature at 50% mass loss (T50%), and the maximum-rate temperature of each degradation stage (Tmax, from the DTG peak) were extracted from the curves. The final residue at 700 °C (char yield) comprises the silica and AR-glass fibers together with any inorganic filler, so an increase in residue with silica content was used as a consistency check on the nominal filler loading.

Fracture surfaces were examined using a CIQTE SEM5000 (CIQTEK, Hefei, China) scanning electron microscope (https://www.ciqtekglobal.com/sem5000 accessed on 29 September 2026) operated at an accelerating voltage of 15 kV. The analyzed surfaces originated from mechanically failed specimens. Areas exhibiting visible fracture features were selected for imaging. The magnification and scale bar are indicated in each micrograph.

The SEM images were analyzed for matrix cracking, fiber pull-out, fiber rupture, fiber–matrix debonding, delamination, matrix retention, void-like features, crack deflection, crack bridging, and clustered regions. This analysis was qualitative, as individual nanoparticles or MWCNTs could not be identified in low-magnification images. Therefore, the SEM images were utilized to compare fracture morphology rather than to assess nanoscale dispersion.

### 2.3. Machine Learning Analysis and Data Treatment

A convolutional neural network (CNN) was employed to analyze the K/G/C–MWCNT dataset. Input variables included MWCNT content, punch load, displacement, tensile strength, and flexural strength. Output variables comprised punch-shear strength, net energy absorption (NEA), and specific energy absorption. The analysis encompassed five MWCNT content levels: 0, 1.5, 3.0, 4.5, and 6.0 wt.%.

The CNN contained three convolutional blocks. ReLU activation and max-pooling operations were applied within the network. Dense layers and a dropout rate of 0.30 followed the convolutional stage. The model used the Adam optimizer with a learning rate of 0.001. Mean squared error was used as the loss function. Early stopping was applied with a patience of 10 epochs, and L2 regularization was set to 0.01. The reported evaluation used nominal five-fold cross-validation.

Punch load and displacement were included as predictors, although these measurements were also used to calculate punch-shear strength and energy absorption. This mathematical dependence creates target leakage and can increase apparent model accuracy. The dataset also contained only five nanofiller levels and no independent manufacturing-batch holdout. Therefore, the CNN results were treated as descriptive curve fitting within the tested formulation range. They were not used to claim externally validated prediction, nanofiller optimization, or performance outside the range of 0–6.0 wt.%.

## 3. Results and Discussion

The subsection results show a consistent concentration-dependent, non-monotonic response across all three laminate architectures. In K/B/ARG- and K/C/ARG-silica laminates, puncture, tensile, flexural, interlaminar shear, and impact properties generally increased with silica content up to 4.5 wt.%, followed by a decline at 6.0 wt.% (Table 3 and Table 4). The K/C/ARG system exhibited particularly significant improvements in puncture, tensile, and flexural performance at 4.5 wt.%. Similarly, K/G/C-MWCNT laminates showed progressive improvements in tensile, ILSS, punch-shear strength, and net energy absorption up to 4.5 wt.%, while flexural strength essentially plateaued and several properties decreased at 6.0 wt.% (Table 5). The SEM observations support these trends, showing greater matrix retention and improved fiber–matrix interaction at intermediate filler contents, whereas higher loading was associated with clustered or weak regions. Overall, the results indicate that intermediate nanofiller contents provided the most favorable mechanical and damage-tolerance response within each investigated laminate architecture.

### 3.1. Mechanical Response and Damage Mechanisms of Silica-Modified K/B/ARG Laminates

#### 3.1.1. Puncture Response and Localized Damage Resistance

In K/B/ARG-silica, the puncture peak load increased from 4270.87 ± 145.21 N at 0 wt.% to 4818.12 ± 149.36 N at 1.5 wt.%, 4958.37 ± 178.50 N at 3.0 wt.%, and 5694.25 ± 187.91 N at 4.5 wt.%, before declining to 5402.25 ± 221.49 N at 6.0 wt.%. The mean value at 4.5 wt.% corresponded to 112.09 ± 3.70 MPa, representing a 33.3% increase over the unmodified mean. This value reflects the highest observed mean, not a statistically validated optimum. The non-monotonic trend aligns with enhanced matrix-mediated load redistribution at intermediate silica loadings and increased viscosity or particle clustering at the highest loading [32,33].

#### 3.1.2. Tensile, Flexural, and Interlaminar Behavior

The tensile strength of K/B/ARG increased from 112.85 ± 3.16 MPa at 0 wt.% to a maximum mean of 167.93 ± 5.71 MPa at 4.5 wt.% (a 48.8% increase), while tensile strain rose from 0.835% to 1.998%. Observed gains at 1.5 and 3.0 wt.% were 8.86% and 17.74%, respectively. The 6.0 wt.% formulation exhibited a decrease relative to 4.5 wt.%. These trends suggest improved stress transfer and delayed matrix-controlled cracking at intermediate silica loadings, with increased heterogeneity at the highest loading.

Flexural strength increased from 87.34 ± 2.62 MPa at 0 wt.% to 109.93 ± 3.85 MPa at 4.5 wt.% (a 25.86% increase), with observed gains of 7.79% and 17.21% at 1.5 and 3.0 wt.%, respectively. The 6.0 wt.% formulation exhibited the highest flexural modulus but not the highest mean flexural strength. This distinction suggests that while additional rigid filler can increase stiffness, clustered or poorly wetted regions may reduce damage tolerance. Therefore, 4.5 wt.% achieved the most favorable balance of stiffness and strength in this system.

Interlaminar shear strength (ILSS) increased from 11.95 ± 0.50 MPa at 0 wt.% to 17.03 ± 0.78 MPa at 4.5 wt.% (a 42.51% increase), then decreased by 9.63% at 6.0 wt.%. Given the matrix and interface sensitivity of ILSS, this trend supports the reinforcement of resin-rich interlayers at intermediate silica loadings [34].

#### 3.1.3. Impact Response and Fracture Mechanisms

The unmodified K/B/ARG laminate exhibited an Izod impact strength of 1036 ± 45.6 J/m. Relative increases at 1.5 and 3.0 wt.% were 22.21% and 31.46%, respectively, with the highest observed mean of 1598.1 ± 73.5 J/m at 4.5 wt.% (54.2% above baseline). Charpy impact strength increased from 266.3 ± 12.3 J/m at 0 wt.% to 526.0 ± 27.4, 623.1 ± 29.9, and 834.9 ± 36.7 J/m at 1.5, 3.0, and 4.5 wt.%, respectively, before declining to 526.9 ± 26.3 J/m at 6.0 wt.%. The response at intermediate loadings is attributed to distributed fiber stretching, matrix cracking, and crack deflection, while the decline at 6.0 wt.% is associated with the formation of more brittle, particle-rich regions.

Fractographic analysis corroborates the mechanical trends, with the better-performing silica condition exhibiting greater matrix retention, increased fiber breakage relative to clean pull-out, more cracking, and reduced fiber–matrix separation. In contrast, the 6.0 wt.% condition displayed weaker matrix regions, increased fiber separation, and more direct fracture paths. These observations support, but do not quantify, a dispersion-dependent change in failure mechanism [35,36].

### 3.2. Mechanical, Thermal, and Fractographic Response of Silica-Modified K/C/ARG Laminates

#### 3.2.1. Puncture Response and Localized Load Transfer

In K/C/ARG-silica, the puncture peak load increased from 4819 ± 168.67 N at 0 wt.% to 5695.57 ± 205.04 N at 1.5 wt.%, 5722.08 ± 240.33 N at 3.0 wt.%, and 7710 ± 285.3 N at 4.5 wt.%, before decreasing to 7460.96 ± 298.44 N at 6.0 wt.%. The highest observed mean was approximately 60.0% above the unmodified mean and represents the maximum within the current K/C/ARG test series, rather than the absolute highest value. This response is architecture-specific, as the outer Kevlar, central carbon, AR-glass interlayers, and modified epoxy collectively influence localized damage [37].

#### 3.2.2. Tensile, Flexural, Interlaminar, and Impact Performance

The tensile strength of K/C/ARG increased from 118.52 ± 3.91 MPa at 0 wt.% to 122.06 ± 3.54, 132.87 ± 4.12, and 171.95 ± 5.50 MPa at 1.5, 3.0, and 4.5 wt.%, respectively, before declining slightly to 167.39 ± 6.86 MPa at 6.0 wt.%. The highest observed mean was 45.1% above the baseline. Tensile strain increased from 1.2% to 1.9% at 4.5 wt.%, then decreased to 1.8% at 6.0 wt.%.

Flexural strength increased from 105.65 ± 3.49 MPa at 0 wt.% to 110.39 ± 4.08, 127.83 ± 4.86, and 157.24 ± 6.45 MPa at 1.5, 3.0, and 4.5 wt.%, respectively, then decreased to 151.8 ± 5.31 MPa at 6.0 wt.%. The mean value at 4.5 wt.% was 48.8% above the baseline. Flexural strain increased from 3.99% at 0 wt.% to 5.1% and 5.4% at 1.5 and 3.0 wt.%, then decreased to 4.6% and 4.4% at 4.5 and 6.0 wt.%. The mismatch between maximum strength and maximum strain is consistent with the combined effects of carbon-fiber stiffness, matrix stiffening, and architecture-dependent stress transfer.

Interlaminar shear strength (ILSS) increased from 13.07 ± 0.55 MPa at 0 wt.% to 14.87 ± 0.68, 18.84 ± 0.83, and 21.69 ± 0.93 MPa at 1.5, 3.0, and 4.5 wt.%, respectively, before declining to 20.51 ± 0.72 MPa at 6.0 wt.%. The mean Izod impact strength increased from 1306.4 ± 52.3 J/m at 0 wt.% to 1362.5 ± 61.3, 1510.0 ± 68.0, and 1813.6 ± 79.8 J/m at 1.5, 3.0, and 4.5 wt.%, respectively, and declined at the highest loading. The 4.5 wt.% formulation thus provided the most favorable balance within this K/C/ARG architecture, although it was not a statistically validated universal optimum [38,39].

#### 3.2.3. Fractographic Evidence for the Silica Optimum

**Figure 1:**
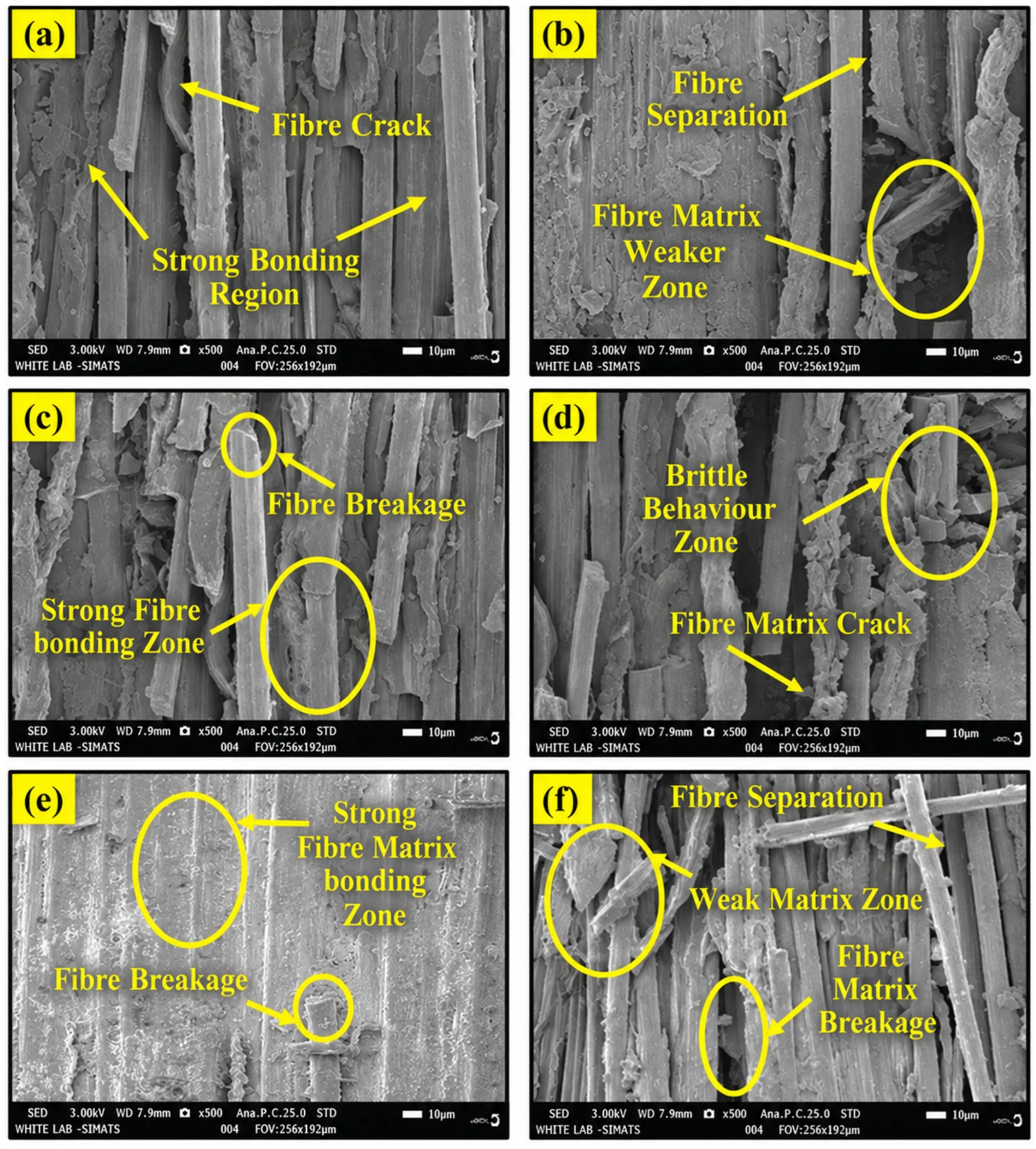
SEM fractographs of silica-modified hybrid laminates: (**a**,**c**,**e**) 4.5 wt.% silica, and (**b**,**d**,**f**) 6.0 wt.% silica. Panels (**a**,**c**,**e**) show greater matrix retention and fiber breakage, whereas panels (**b**,**d**,**f**) show fiber separation, matrix cracking, and weak regions.

Figure 1 presents representative fracture regions for the 4.5 and 6.0 wt.% silica conditions. Panels (a), (c), and (e) demonstrate stronger matrix retention and increased fiber breakage in the better-performing condition, while panels (b), (d), and (f) illustrate fiber separation, matrix cracking, and weak regions at 6.0 wt.%. These qualitative features suggest that greater energy was dissipated through fiber fracture, matrix deformation, and crack deflection at intermediate loading. However, these observations do not directly quantify particle dispersion. This interpretation aligns with previous studies indicating that dispersed nanosilica can enhance epoxy fracture response, whereas increased viscosity and agglomeration restrict effective loading [5,12,14].

#### 3.2.4. Thermal Stability and Mechanical–Thermal Correlation

**Figure 2:**
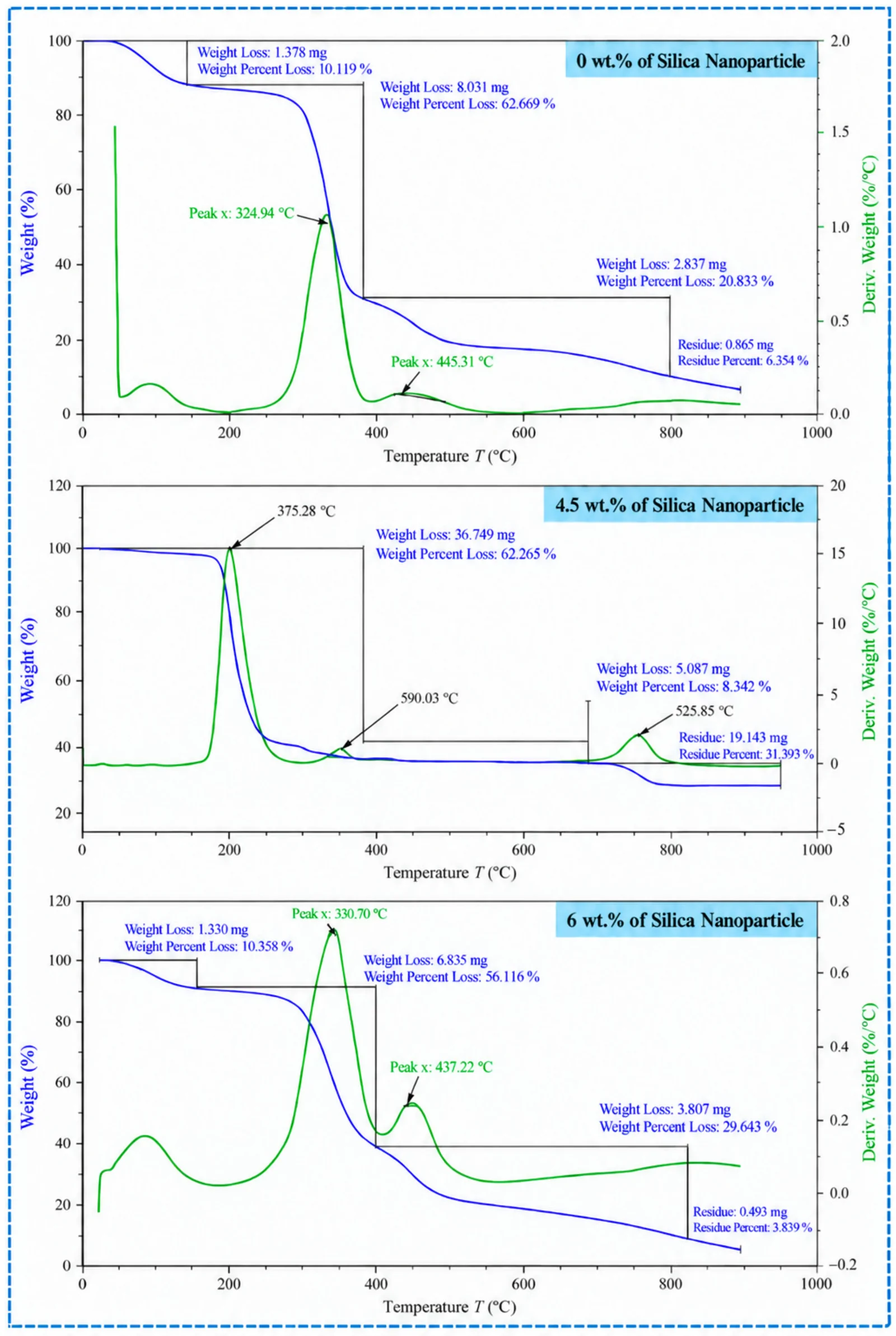
Thermogravimetric (blue, mass) and derivative mass-loss (green, DTG) traces for K/C/ARG laminates containing 0, 4.5, and 6.0 wt.% silica.

Figure 2 presents the thermogravimetric analysis (TGA) and derivative thermogravimetry (DTG) responses of K/C/ARG laminates containing 0, 4.5, and 6.0 wt.% silica. The blue curves indicate the remaining mass, while the green curves represent the mass-loss rate. All laminates demonstrate multistage degradation, including moisture or volatile removal, epoxy-network decomposition, organic-fiber degradation, and the formation of char and inorganic residue. Comparable behavior has been reported for silica-modified epoxy systems [40].

The unmodified laminate exhibits initial and major mass losses of 10.119% and 62.669%, respectively, with DTG features at 324.94 and 445.31 °C and a final residue of 6.354%. The initial loss is attributed to moisture and volatile constituents, while the principal stage corresponds to epoxy decomposition and subsequent degradation of the organic reinforcement. For the laminate with 4.5 wt.% silica, the main DTG feature shifts to 375.28 °C, approximately 50 °C higher than that of the unmodified laminate, with additional features at 525.85 and 590.03 °C. The observed residue is 19.143 mg, or 31.393% of the initial mass. The shift in the principal DTG temperature and the increased residue indicate improved condensed-phase stability. Well-dispersed silica restricts polymer-chain mobility, hinders volatile transport, and promotes the formation of thermally persistent silica-rich or carbonaceous residue [12,40]. This behavior aligns with the superior mechanical performance and enhanced matrix retention observed at 4.5 wt.%, although it does not confirm a common mechanism. At 6.0 wt.% silica, the principal DTG features occur at 330.70 and 437.22 °C, while the residue decreases to 3.839%. Therefore, increasing silica content beyond 4.5 wt.% does not further improve the thermal response. Higher nanoparticle contents increase resin viscosity, promote agglomeration, hinder fiber wetting, and reduce the effective interfacial area [12,30]. This interpretation is supported by the clustered and weak regions observed in the corresponding scanning electron microscopy (SEM) image.

In construction applications, delayed maximum-rate degradation and increased residue can help maintain a protective condensed phase during short-term thermal exposure. However, TGA alone does not establish fire resistance, flame spread, smoke generation, or structural performance during fire. Additionally, only one TGA run was conducted per composition (n = 1); the reported mass changes for the 4.5 wt.% specimen do not sum precisely to 100%, and some labels are misaligned on the temperature axis.

### 3.3. Mechanical Response, Energy Absorption, and CNN Prediction of MWCNT-Modified K/G/C Laminates

#### 3.3.1. Tensile, Flexural, and Interlaminar Response

In K/G/C-MWCNT composites, tensile strength increased from 136.35 ± 4.23 MPa at 0 wt.% to 199.50 ± 6.78, 202.53 ± 6.48, and 215.58 ± 7.76 MPa at 1.5, 3.0, and 4.5 wt.%, respectively, before slightly decreasing to 213.42 ± 6.19 MPa at 6.0 wt.%. Tensile strain rose from 3.2% to 6.0% between 0 and 4.5 wt.% and then declined to 5.5% at 6.0 wt.%. Tensile modulus increased from 20,508.27 MPa to 28,042.76 MPa, followed by a decrease to 27,331.10 MPa. Although the 4.5 wt.% level yielded the highest observed means, the small differences near the maximum cannot be considered statistically significant in the absence of replicate-level data.

Flexural strength measured 294.35 ± 10.30 MPa at 0 wt.%, 296.67 ± 11.57 MPa at 1.5 wt.%, 308.08 ± 9.86 MPa at 3.0 wt.%, 320.18 ± 13.13 MPa at 4.5 wt.%, and 320.02 ± 11.52 MPa at 6.0 wt.%. The nearly identical values at 4.5 and 6.0 wt.% suggest a plateau rather than a decrease. Interlaminar shear strength (ILSS) exhibited greater sensitivity, increasing from 18.84 ± 0.90 MPa at 0 wt.% to 20.51 ± 0.94, 22.25 ± 1.00, and 28.49 ± 1.20 MPa at 1.5, 3.0, and 4.5 wt.%, respectively, before decreasing to 23.91 ± 1.22 MPa at 6.0 wt.%. Crack bridging delays matrix-crack opening, while nanotube debonding and frictional pull-out dissipate energy. In contrast, clustering reduces the number of effective nanotubes and introduces weak regions [37,41,42].

#### 3.3.2. Punch-Shear Response and Energy Absorption

Punch-shear load and strength increased from 3385.52 N and 88.85 ± 4.44 MPa at 0 wt.% to 3427.80 N and 89.96 ± 4.49 MPa at 1.5 wt.%, 3762.60 N and 98.75 ± 4.93 MPa at 3.0 wt.%, and 4221.60 N and 110.80 ± 5.54 MPa at 4.5 wt.%, then decreased to 4072.26 N and 106.89 ± 5.34 MPa at 6.0 wt.% (n = 3). The overlapping uncertainty ranges at 4.5 and 6.0 wt.% indicate that the difference is not statistically significant. The 45° Kevlar/carbon layers redistribute stress around the shear zone, the 0° glass layer provides in-plane stability, and the modified matrix carries local interlaminar stress.

NEA increased from 3.28 ± 0.16 J at 0 wt.% to 7.31 ± 0.36, 10.88 ± 0.54, and 15.45 ± 0.77 J at 1.5, 3.0, and 4.5 wt.%, respectively, before decreasing to 13.05 ± 0.65 J at 6.0 wt.% (n = 3). Specific energy absorption increased from 0.10 ± 0.005 J/g to 0.19 ± 0.009, 0.32 ± 0.01, 0.44 ± 0.02, and 0.39 ± 0.01 J/g. The non-overlapping uncertainty ranges for NEA at 4.5 and 6.0 wt.% indicate a clear difference. This trend aligns with changes in crack bridging, pull-out, deflection, interfacial friction, delamination, and distributed matrix failure [6].

#### 3.3.3. SEM Evidence and Damage Evolution

**Figure 3:**
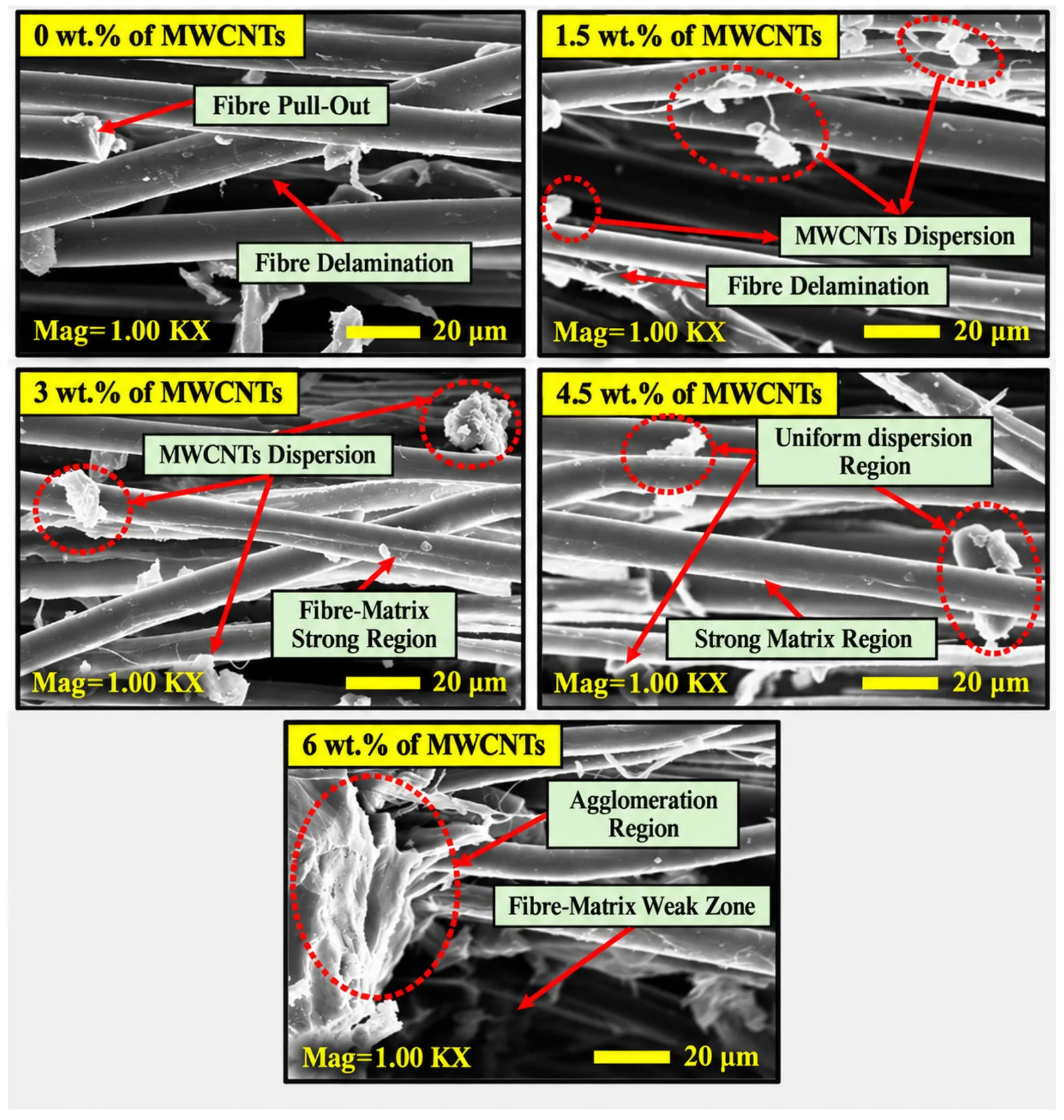
SEM fields across MWCNT contents: clean fiber pull-out with weak fiber–matrix adhesion at 0 wt.%, more continuous interfacial regions at intermediate contents, and a clustered weak region at 6.0 wt.%.

Figure 3 displays SEM images of fractured K/G/C laminates containing 0–6.0 wt.% MWCNTs. The unmodified laminate exhibits clean exposed fibers, extensive fiber pull-out, and interfacial delamination, indicating weak fiber–matrix adhesion and preferential crack propagation along the interface. The limited matrix remaining on the fibers corresponds with the comparatively low tensile strength, interlaminar shear strength (ILSS), punch-shear resistance, and net energy absorption. At 1.5 wt.% MWCNTs, increased matrix retention is observed on the fibers, despite localized delamination. This observation suggests enhanced interfacial engagement. MWCNTs are likely to restrict crack opening and dissipate energy through debonding, frictional sliding, and pull-out, which may explain the initial improvement in tensile strength and energy absorption [17,19].

At 3.0 wt.% MWCNTs, the micrograph reveals more continuous matrix coverage and fewer completely exposed fibers, which suggests improved stress transfer and a transition from adhesive interfacial separation to more cohesive matrix damage. Crack deflection and nanotube bridging are likely to further increase the energy required for damage propagation. This interpretation is supported by increases in tensile strength from 136.35 ± 4.23 to 202.53 ± 6.48 MPa, ILSS from 18.84 ± 0.90 to 22.25 ± 1.00 MPa, punch-shear strength from 88.85 ± 4.44 to 98.75 ± 4.93 MPa, and net energy absorption from 3.28 ± 0.16 to 10.88 ± 0.54 J. Furthermore, the 4.5 wt.% laminate demonstrates the most continuous matrix-rich regions and the strongest matrix retention around the fibers. The presence of fewer cleanly pulled-out fibers and large interfacial gaps indicates more effective load transfer and greater resistance to delamination. A well-distributed MWCNT network can bridge microcracks, increase crack-path tortuosity, and dissipate energy through debonding and pull-out. These mechanisms are consistent with the observed maximum tensile strength of 215.58 ± 7.76 MPa, flexural strength of 320.18 ± 13.13 MPa, ILSS of 28.49 ± 1.20 MPa, punch-shear strength of 110.80 ± 5.54 MPa, and net energy absorption of 15.45 ± 0.77 J. Therefore, 4.5 wt.% represents the best-performing MWCNT content tested in this study. However, this value should not be regarded as a universally applicable optimum.

At 6.0 wt.% MWCNTs, a large clustered matrix-rich feature is observed adjacent to an apparent fiber–matrix weak zone. Elevated nanotube contents can increase resin viscosity, hinder fiber wetting, and promote clustering, entrapped air, and resin-rich regions. These defects generate stress concentrations and reduce the effective load-transfer area. Consequently, ILSS decreased from 28.49 ± 1.20 to 23.91 ± 1.22 MPa, punch-shear strength from 110.80 ± 5.54 to 106.89 ± 5.34 MPa, and net energy absorption from 15.45 ± 0.77 to 13.05 ± 0.65 J. The nearly unchanged flexural strengths at 4.5 and 6.0 wt.% indicate a plateau rather than substantial flexural deterioration. Previous studies have reported similar concentration-dependent effects of nanotube dispersion and clustering [41,42]. However, the alumina matrix and processing route in those studies differ from the present epoxy laminate, so the comparison is mechanistic rather than quantitative.

#### 3.3.4. CNN Prediction, Error Structure, and Model Limitations

**Figure 4:**
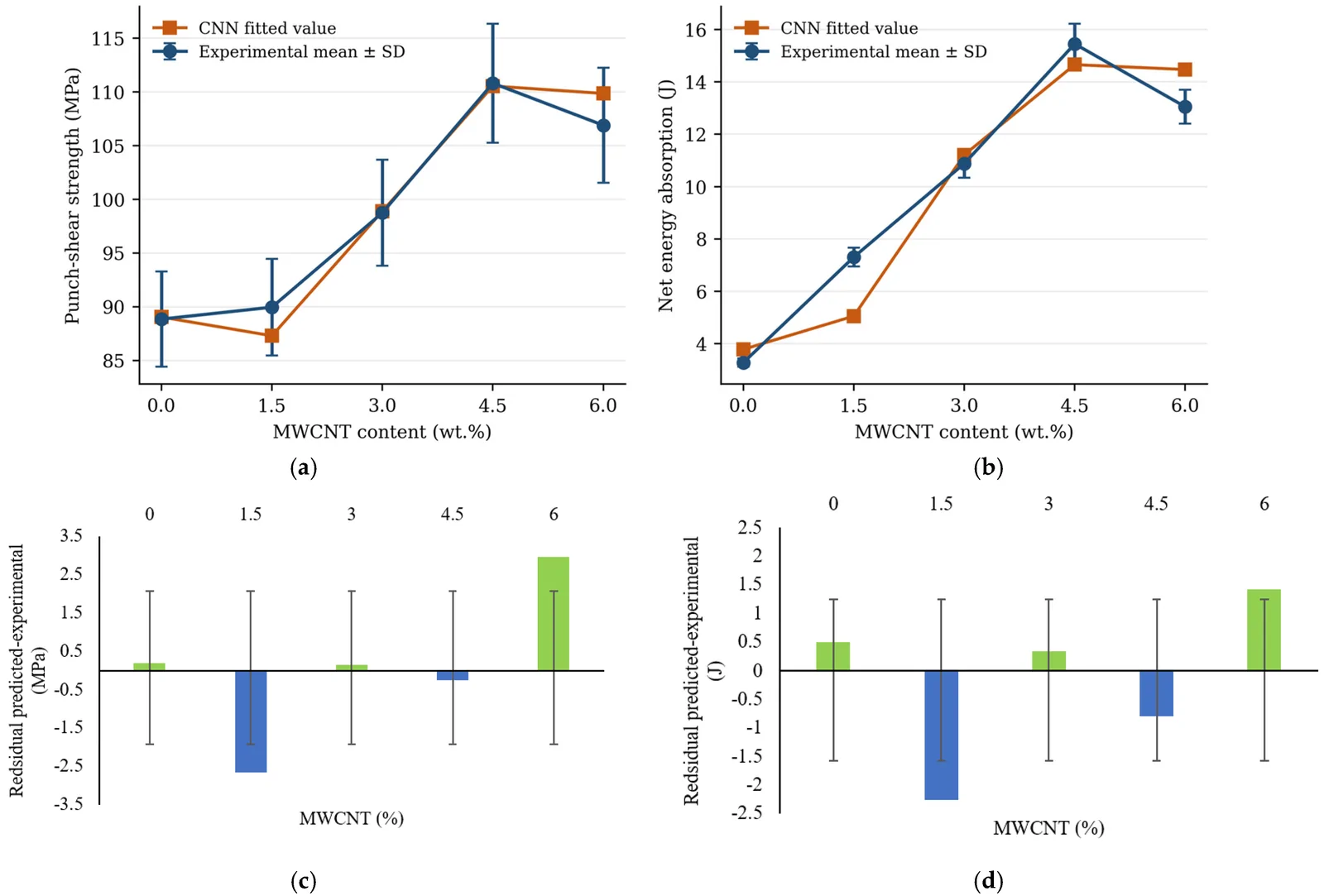
Experimental means and SD error bars (n = 3), CNN-fitted values, and signed residuals for (**a**,**c**) punch-shear strength and (**b**,**d**) net energy absorption.

Figure 4 presents a comparison between experimental means and CNN-fitted values. For punch-shear strength, the fitted minus experimental residuals were +0.194, −2.662, +0.142, −0.257, and +2.961 MPa at 0, 1.5, 3.0, 4.5, and 6.0 wt.%, respectively. Experimental error bars indicate the SD values for n = 3, while the CNN curve lacks an independently estimated predictive interval. The largest positive strength residual was observed at 6.0 wt.%. However, attributing this error exclusively to microstructural heterogeneity remains speculative.

For NEA, the fitted minus experimental residuals were +0.506, −2.253, +0.333, −0.793, and +1.420 J. The residuals change sign across the formulation range and reach their largest magnitude at 1.5 wt.%, indicating that the fitted curve does not reproduce all concentration levels with equal accuracy. Energy absorption is influenced by multiple stochastic damage processes that are not fully captured by nanofiller content and correlated mechanical predictors alone.

The reported in-sample and cross-validation summary metrics were as follows: MAE = 1.2430 MPa, RMSE = 1.7874 MPa, and R^2^ = 0.9876 for punch-shear strength; MAE = 1.0608 J, RMSE = 1.2716 J, and R^2^ = 0.9654 for NEA; and MAE = 0.0224 J/g, RMSE = 0.0289 J/g, and R^2^ = 0.9987 for specific energy absorption. These metrics reflect interpolation within a single, small formulation family. Due to the availability of only five formulation levels, the absence of retained fold assignments, and the inclusion of mechanically related outcomes from the same experimental program as predictors, these values do not establish external predictive validity [25,43].

### 3.4. Interpretation of Structure–Property–Damage Relationships, Design Implications, and Study Limitations

#### 3.4.1. Influence of Architectural Parameters on Mechanical Performance at the Standard 4.5 wt.% Loading

**Figure 5:**
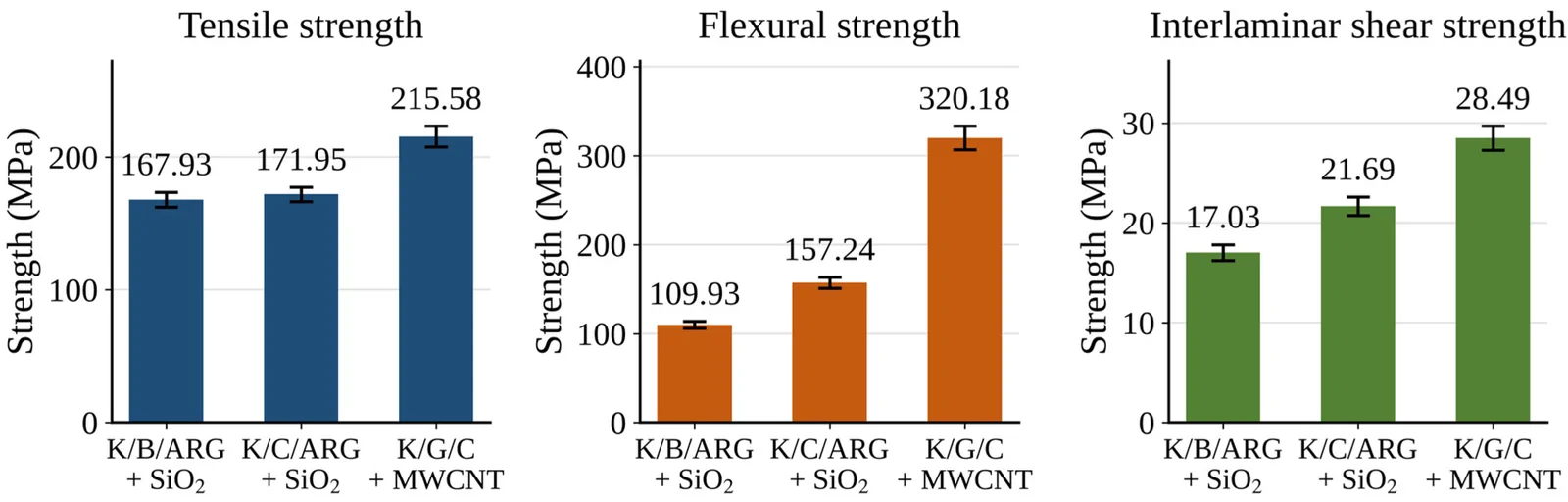
Architecture-specific tensile, flexural, and interlaminar-shear strength means at the common 4.5 wt.% tested level. Error bars denote the standard deviation (n = 3).

Figure 5 presents the results at the standardized 4.5 wt.% loading. The observed differences among systems arise from concurrent variations in fiber type, ply count, stacking sequence, nanofiller type, and, for localized loading, test configuration. These results should not be interpreted as a direct comparison between silica and MWCNTs. Within each material family, the 4.5 wt.% loading produced the highest mean values for tensile strength, interlaminar shear strength (ILSS), and most other measured responses. Notably, the K/G/C system exhibited similar flexural strength at both 4.5 wt.% (320.18 MPa) and 6.0 wt.% (320.02 MPa).

The K/G/C-MWCNT laminate demonstrated higher absolute mean values for tensile, flexural, and ILSS compared to the silica-modified systems; however, these differences cannot be solely attributed to the presence of MWCNTs. Variations in carbon content, fiber placement, orientation, adjacent glass or AR-glass layers, and ply count influence the principal stress distribution. Consequently, this comparison serves to elucidate architecture-specific response patterns and matrix-sensitive failure modes, rather than establishing a ranking of nanofiller effectiveness [43,44,45].

#### 3.4.2. Localized Resistance, Impact, and Energy-Absorption Response

**Figure 6:**
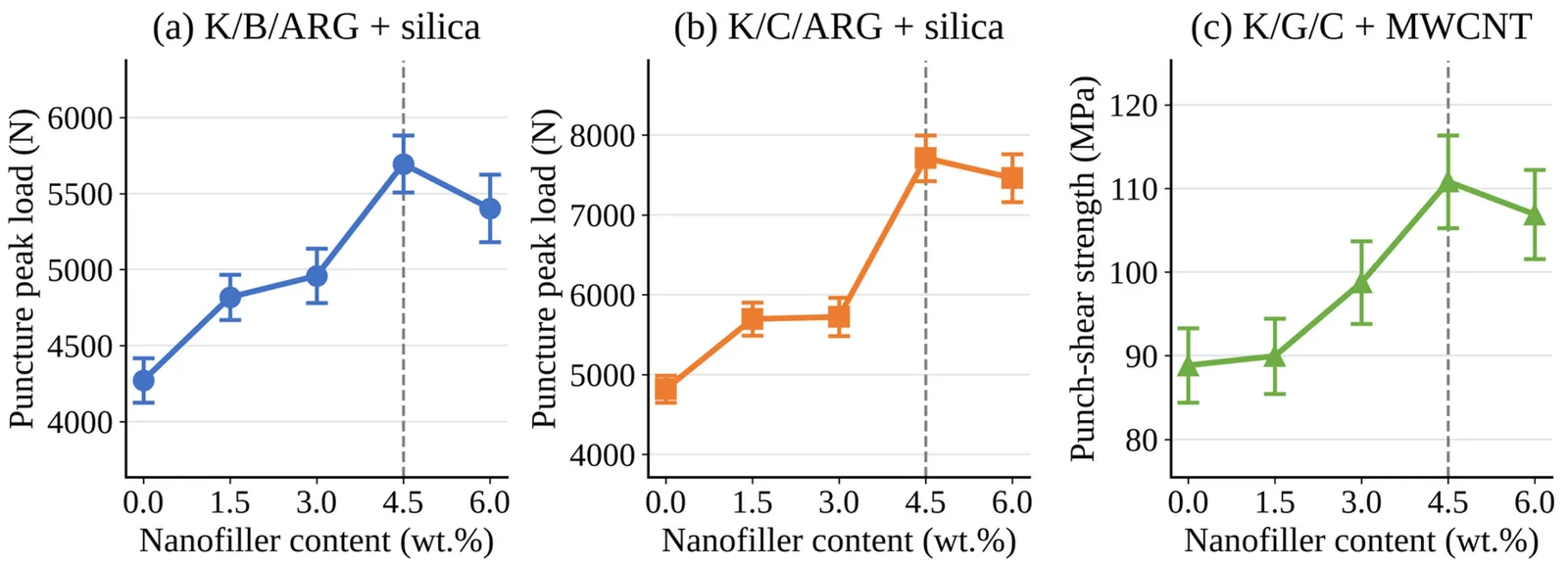
Architecture-specific localized resistance shown in separate panels: (**a**) K/B/ARG-silica puncture peak load, (**b**) K/C/ARG-silica puncture peak load, and (**c**) K/G/C-MWCNT ASTM D732 punch-shear strength. Error bars denote the standard deviation (n = 3) in all panels.

Figure 6 distinguishes the two-puncture series from the ASTM D732 punch-shear series. In all three cases, the highest observed mean occurs at 4.5 wt.%, followed by a lower or similar response at 6.0 wt.%. However, the absolute values are not directly comparable due to differences in specimen geometry, boundary conditions, contact, stress field, and reported response. The vertical dashed line at 4.5 wt.% indicates the concentration of the observed maxima but does not represent a statistically established universal optimum.

In this study, K/C/ARG-silica achieved a puncture peak load of 7710 ± 285.3 N, K/B/ARG-silica achieved 5694.25 ± 187.91 N and 112.09 MPa puncture strength, and K/G/C-MWCNT achieved 4221.6 N and 110.80 ± 5.54 MPa punch-shear strength. Impact and absorbed-energy values were obtained using different procedures and units: 1598.1 ± 73.5 J/m Izod and 834.9 ± 36.7 J/m Charpy for K/B/ARG-silica; 1813.6 ± 79.8 J/m Izod for K/C/ARG-silica; and 15.45 ± 0.77 J NEA and 0.44 ± 0.02 J/g for K/G/C-MWCNT. These values should be interpreted within the context of their respective methods and are not directly comparable across tests.

#### 3.4.3. Common Processing–Structure–Property Mechanism

Among the three independent laminate families, both mechanical performance and qualitative fracture morphology were most favorable at intermediate loading levels. Dispersed silica likely stiffens resin-rich regions, deflects cracks, and enhances interfacial stress transfer, while individual MWCNTs may bridge cracks and dissipate energy through debonding, friction, and pull-out. These mechanisms align with the observed trends, although they were not directly measured using quantitative dispersion mapping [19,34,42,46].

At 6.0 wt.%, several responses plateaued or declined, and SEM analysis revealed clustered or weak regions. Increased filler content can raise viscosity, hinder fiber wetting, promote void retention, and reduce the effective reinforcement surface area. Yamamoto et al. [42] demonstrated that dispersion state significantly affects nanotube-composite properties, and the present epoxy results similarly associate clustered morphology with reduced ILSS and energy absorption. Since the current SEM assessment is qualitative, the proposed mechanism is supported by the convergence of observed trends rather than quantitative proof [14,30].

#### 3.4.4. Limitations and Design Implications

The primary limitation of this study stems from variations in fiber type, stacking sequence, nanofiller type, and testing protocols across systems. As a result, the study supports within-family concentration trends but does not enable a causal comparison between silica and MWCNTs. Most conditions used n = 3, and specimen-level results, confidence intervals, and formal significance tests were not retained except for selected MWCNT punch-shear and energy measures. SEM analysis was qualitative and did not yield dispersion indices, void fraction, or compositional mapping. Additionally, the CNN was trained on a single, internally related dataset and lacks demonstrated external validity.

Future research should maintain constant laminate architecture while varying nanofiller type, increase independent batch replication, retain raw specimen-level data, and quantify fiber and void volume fractions, rheology, cure state, and dispersion. Testing should also focus on narrower concentration ranges around 4.5 wt.%. Machine learning evaluation should incorporate batch-wise holdout data, simpler baseline models, uncertainty intervals, and microstructural descriptors. These steps are essential to distinguish between a sharp maximum and a broad performance plateau, and to advance from descriptive trends to statistically supported material design.

## 4. Conclusions

In this study, the concentration-dependent responses were evaluated within three hybrid epoxy laminate families. The findings are specific to the tested materials, stacking sequences, fabrication conditions, and test configurations.

Within each laminate family, 4.5 wt.% nanofiller yielded the highest mean values for most measured properties. Several responses declined or plateaued at 6.0 wt.%. Thus, 4.5 wt.% is identified as the best-performing tested concentration, rather than a statistically established universal optimum.

At 4.5 wt.% silica, the K/B/ARG laminate exhibited 167.93 ± 5.71 MPa tensile strength, 109.93 ± 3.85 MPa flexural strength, 17.03 ± 0.78 MPa ILSS, 112.09 ± 3.70 MPa puncture strength, 1598.1 ± 73.5 J/m Izod impact strength, and 834.9 ± 36.7 J/m Charpy impact strength. Compared to the unmodified laminate, tensile, flexural, ILSS, puncture, and Izod strengths increased by 48.8%, 25.9%, 42.5%, 33.3%, and 54.2%, respectively.

At 4.5 wt.% silica, the K/C/ARG laminate achieved 171.95 ± 5.50 MPa tensile strength, 157.24 ± 6.45 MPa flexural strength, 21.69 ± 0.93 MPa ILSS, 7710 ± 285.3 N puncture load, and 1813.6 ± 79.8 J/m Izod impact strength. These values represent the highest observed means within the K/C/ARG series. At 6.0 wt.%, the corresponding tensile, flexural, ILSS, and puncture responses decreased.

At 4.5 wt.% MWCNTs, the K/G/C laminate demonstrated 215.58 ± 7.76 MPa tensile strength, 320.18 ± 13.13 MPa flexural strength, 28.49 ± 1.20 MPa ILSS, 110.80 ± 5.54 MPa punch-shear strength, and 15.45 ± 0.77 J net energy absorption. At 6.0 wt.%, ILSS, punch-shear strength, and net energy absorption decreased to 23.91 ± 1.22 MPa, 106.89 ± 5.34 MPa, and 13.05 ± 0.65 J, respectively. Flexural strength remained nearly constant at 320.02 ± 11.52 MPa.

SEM observations indicated greater matrix retention, fewer cleanly pulled-out fibers, and more distributed fracture at intermediate nanofiller contents. The 6.0 wt.% specimens exhibited clustered matrix-rich or weak regions. These images support a concentration-dependent change in fracture morphology but do not quantify nanoparticle or MWCNT dispersion.

For K/C/ARG, the single TGA run at 4.5 wt.% silica revealed a principal DTG feature at 375.28 °C and a reported residue of 31.393%. The corresponding control values were 324.94 °C and 6.354%. These results suggest improved condensed-phase retention in the tested specimen. However, the thermal finding requires replicate TGA measurements and verification of the plotted labels and mass normalization. TGA alone does not demonstrate fire resistance.

The CNN reproduced trends within the five K/G/C–MWCNT formulation levels. However, it did not provide independently validated predictions due to the small dataset, mathematical relationships between punch load, displacement, and target variables, and the absence of an independent batch-level holdout. Future modeling should utilize raw specimen-level data, batch-wise validation, uncertainty intervals, and comparison with simpler baseline models.

The findings support the development of hybrid laminates for use in protective panels, strengthening systems, and components subjected to localized loading. Prior to structural application, further research should verify fiber and void volume fractions, nanofiller dispersion, durability, fire behavior, and full-scale performance. Future experiments should maintain a constant laminate architecture while directly comparing silica and MWCNTs across replicated manufacturing batches.

## Figures and Tables

**Table 1:** Laminate architectures, nanofiller levels, and experimental scope.

System	Nano Modifier	Stacking Sequence	Nanofiller (wt.%)	Principal Evaluations
I: K/B/ARG	Silica	0°K2/45°B2/0°ARG2/45°B2/0°K2	0, 1.5, 3.0, 4.5, 6.0	Tensile, flexural, ILSS, puncture, Izod/Charpy, SEM
II: K/C/ARG	Silica	0°K2/45°ARG2/0 °C2/45°ARG2/0°K2	0, 1.5, 3.0, 4.5, 6.0	Tensile, flexural, ILSS, puncture, Izod, TGA, SEM
III: K/G/C	MWCNT	45°K/45 °C/0°G/45 °C/45°K	0, 1.5, 3.0, 4.5, 6.0	Tensile, flexural, ILSS, punch shear, energy absorption, SEM, CNN

**Table 2:** Mechanical tests and specimen dimensions used in the experimental program.

Test	Standard/Procedure	Nominal Specimen Size (mm)	Primary Response
Tensile	ASTM D3039/D3039M-17; 2.0 mm min^−1^	250 × 25 × 4	Strength, strain, modulus
Flexural	ASTM D790-17; 64 mm span; 1.7 mm min^−1^	127 × 12.7 × 4	Flexural strength, strain, modulus
Interlaminar shear	ASTM D2344/D2344M-16; 16 mm span; 1.0 mm min^−1^	40 × 10 × 4	ILSS
Quasi-static puncture	ASTM D6264-type QSI; 12.7 mm hemispherical indenter; 1.0 mm min^−1^	90 × 90 × 4	Peak load/puncture strength
Punch shear	ASTM D732-17; 25.4 mm punch; 1.25 mm min^−1^	50 × 50 × 4	Peak load/punch-shear strength
Izod impact	ASTM D256; pendulum impact	64 × 12.7 × 4	Impact strength
Charpy impact	ASTM D6110; pendulum impact	127 × 12.7 × 4	Impact strength

**Table 3:** Concentration-level means ± SD for K/B/ARG-silica (n = 3).

Silica (wt.%)	Puncture Load (N)	Tensile (MPa)	Flexural (MPa)	ILSS (MPa)	Izod (J/m)	Charpy (J/m)
0	4270.87 ± 145.21	112.85 ± 3.16	87.34 ± 2.62	11.95 ± 0.50	1036.0 ± 45.6	266.3 ± 12.3
1.5	4818.12 ± 149.36	122.85 ± 4.05	94.15 ± 3.20	14.62 ± 0.66	1266.1 ± 60.8	526.0 ± 27.4
3	4958.37 ± 178.50	132.87 ± 4.12	102.38 ± 2.97	15.97 ± 0.74	1361.9 ± 57.2	623.1 ± 29.9
4.5	5694.25 ± 187.91	167.93 ± 5.71	109.93 ± 3.85	17.03 ± 0.78	1598.1 ± 73.5	834.9 ± 36.7
6	5402.25 ± 221.49	158.46 ± 5.42	106.11 ± 3.79	15.39 ± 0.63	1476.2 ± 66.4	526.9 ± 26.3

**Table 4:** Concentration-level means ± SD for K/C/ARG-silica (n = 3).

Silica (wt.%)	Puncture Load (N)	Tensile (MPa)	Flexural (MPa)	ILSS (MPa)	Izod (J/m)
0	4819.00 ± 168.67	118.52 ± 3.91	105.65 ± 3.49	13.07 ± 0.55	1306.4 ± 52.3
1.5	5695.57 ± 205.04	122.06 ± 3.54	110.39 ± 4.08	14.87 ± 0.68	1362.5 ± 61.3
3	5722.08 ± 240.33	132.87 ± 4.12	127.83 ± 4.86	18.84 ± 0.83	1510.0 ± 68.0
4.5	7710.00 ± 285.3	171.95 ± 5.50	157.24 ± 6.45	21.69 ± 0.93	1813.6 ± 79.8
6	7460.96 ± 298.44	167.39 ± 6.86	151.80 ± 5.31	20.51 ± 0.72	1702.5 ± 76.6

**Table 5:** Concentration-level results for K/G/C-MWCNT (n = 3; mean ± SD).

MWCNT (wt.%)	Tensile (MPa)	Flexural (MPa)	ILSS (MPa)	Punch Shear (MPa)	NEA (J)
0	136.35 ± 4.23	294.35 ± 10.30	18.84 ± 0.90	88.85 ± 4.44	3.28 ± 0.16
1.5	199.50 ± 6.78	296.67 ± 11.57	20.51 ± 0.94	89.96 ± 4.49	7.31 ± 0.36
3	202.53 ± 6.48	308.08 ± 9.86	22.25 ± 1.00	98.75 ± 4.93	10.88 ± 0.54
4.5	215.58 ± 7.76	320.18 ± 13.13	28.49 ± 1.20	110.80 ± 5.54	15.45 ± 0.77
6	213.42 ± 6.19	320.02 ± 11.52	23.91 ± 1.22	106.89 ± 5.34	13.05 ± 0.65

## Data Availability

The data supporting the findings of this study are presented within the article. Additional information regarding the reported results may be obtained from the corresponding author upon reasonable request.
